# Demographic history and population genetic analysis of *Decapterus maruadsi* from the northern South China Sea based on mitochondrial control region sequence

**DOI:** 10.7717/peerj.7953

**Published:** 2019-10-28

**Authors:** Su-Fang Niu, Ren-Xie Wu, Yun Zhai, Hao-Ran Zhang, Zhong-Lu Li, Zhen-Bang Liang, Yu-Hang Chen

**Affiliations:** College of Fisheries, Guangdong Ocean University, Zhanjiang, Guangdong, China

**Keywords:** *Decapterus maruadsi*, Demographic history, Population genetic, The northern South China Sea

## Abstract

Late Pleistocene climate oscillations are believed to have greatly influenced the distribution, population dynamics, and genetic variation of many marine organisms in the western Pacific. However, the impact of the late Pleistocene climate cycles on the demographic history and population genetics of pelagic fish in the northern South China Sea (SCS) remains largely unexplored. In this study, we explored the demographic history, genetic structure, and genetic diversity of *Decapterus maruadsi*, a typical pelagic fish, over most of its range in the northern SCS. A 828–832 bp fragment of mitochondrial control region were sequenced in 241 individuals from 11 locations. High haplotype diversity (0.905–0.980) and low nucleotide diversity (0.00269–0.00849) was detected, revealing low levels of genetic diversity. Demographic history analysis revealed a pattern of decline and subsequent rapid growth in the effective population size during deglaciation, which showed that *D. maruadsi* experienced recent demographic expansion after a period of low effective population size. Genetic diversity, genetic structure, and phylogenetic relationship analysis all demonstrated that no significant genetic differentiation existed among the populations, indicating that *D. maruadsi* was panmictic throughout the northern SCS. Periodic sea-level changes, fluctuation of the East Asian Monsoon, and Kuroshio variability were responsible for the population decline and expansion of *D. maruadsi.* The demographic history was the primary reason for the low levels of genetic diversity and the lack of significant genetic structure. The life history characteristics and ocean currents also had a strong correlation with the genetic homogeneity of *D. maruadsi*. However, the genetic structure of the population (genetic homogeneity) is inconsistent with biological characteristics (significant difference), which is an important reminder to identify and manage the *D. maruadsi* population carefully.

## Introduction

Late Pleistocene climate oscillations drastically altered the sea-level and nutrient inputs into the western Pacific marginal seas. This is believed to have greatly influenced the population distribution, genetic variation, and demographic history of marine organisms (Hewitt, 2000; Lambeck, Esat & Potter, 2002; Liu et al., 2007; Xue et al., 2014; He et al., 2015). For example, the South China Sea (SCS) became a semi-enclosed sac-shaped gulf and a total of 0.7 million km^2^ of continental shelf was exposed during the Last Glacial Maximum (LGM, 26.5–19.0 kyr before present) (Wang & Sun, 1994; Clark et al., 2009). The remarkable changes would have greatly reduced the available habitat for marine organisms, and the displaced populations would have likely retreated to the glacial refuge of the SCS during the LGM (Liu et al., 2006; He et al., 2014). Meanwhile, the LGM also unquestionably changed the surface circulation in the SCS, with the eastward shift of the Kuroshio path, for example (Nan, Xue & Yu, 2015). Nutrient inputs changed along with the East Asian Monsoon circulations and sea-level fluctuations, which are closely associated with primary productivity and fish production (Jian, Wang & Kienast, 1999; Jian et al., 1999). Thus, the cyclic changes during the Late Pleistocene era in the sea-level and nutrient inputs likely led to expansions and contractions in the populations of marine organisms (Grant & Bowen, 1998; Liu et al., 2006; He et al., 2014). These historic processes are thought to have greatly influenced the evolutionary patterns of the populations of several coastal organisms in the northern SCS, such as genetic differentiation caused by population contraction or genetic homogeneity resulting from population expansion (Liu et al., 2007; He et al., 2010; He et al., 2015). However, the impact of late Pleistocene climatic cycles on the demographic history and population genetics of pelagic fish in the northern SCS remains largely unexplored.

The round scad, *Decapterus maruadsi* (Temminck & Schlegel, 1844), is a warm-water pelagic fish of the family Carangidae (Teleostei, Perciformes). It is widely distributed in the coastal waters of the western Pacific, including those near China, Korea, and Japan (Liu et al., 2016). It is one of the most important fish in China and is especially abundant in the northern SCS (Deng & Zhao, 1991). The annual catch from the northern SCS reached about 2. 8 ×10^5^ tons between 1998–2017, with a maximum catch of approximately 3. 6 ×10^5^ tons being recorded in 2002 (Ministry of Agriculture Fishery Administration, 1999-2018). In the northern SCS, *D. maruadsi* is endemic and has obvious regional characteristics (Deng & Zhao, 1991). The fish is not a long-distance migration species and only migrates seasonally over short distances from North-South or deep-shallow in the continental shelf (<180 m) of the northern SCS (Zheng et al., 2014). Moreover, *D. maruadsi* spawns mainly in the northern shallow continental shelf of the SCS (less than 120 m). The exposure of the shallow continental shelf of the northern SCS during the late Pleistocene climate fluctuations would inevitably change the population abundance, demographic history, and genetic variation of *D. maruadsi*. For these reasons, *D. maruadsi* is a good fish with which to research the effects of paleoclimatic fluctuations on the demographic history and population genetics of pelagic fish in the northern SCS.

The previous studies of *D. maruadsi* have mainly focused on resource and fishery biology and little is known about their demographic history. Although the population genetics of *D. maruadsi* in the northern SCS have been analyzed by mitochondrial cytochrome *b* (Cyt *b*) sequence variation (Niu et al., 2018), the results of population genetic homogeneity are not consistent with those of the demographic structure. According to its distribution and ecological characteristics, *D. maruadsi* in the northern SCS is divided into seven different stocks, including the Minnan-Taiwan shoal, the area off Jiazi in the east Guangdong, the Pearl River estuary, western Guangdong, Hainan Qinglan, Beibu Gulf, and the sea off of the Pearl River estuary (Deng & Zhao, 1991). These inconsistencies require us to determine the population structure of *D. maruadsi* in the northern SCS using a mitochondrial control region sequence that has a faster evolutionary rate than Cyt *b*. Accurate genetic data is necessary to appropriately identify the ‘Management Units’ (MUs), which are fundamental to proper short-term management (Moritz, 1994). Genetic data has been widely used recently in the identification of the populations and conservation units for some aquatic species (Waples, Punt & Cope, 2008).

In the present study, 11 populations of *D. maruadsi* were collected throughout the northern SCS to assess the evolutionary history and population genetics based on the mitochondrial control region sequence. Specifically, we attempted to investigate the evolutionary influences of Late Pleistocene era sea-level fluctuations, monsoon oscillations, and the shift of surface circulation on the demographic history and genetic variation of *D. maruadsi*. The results will provide new insights into the MU’s identification and consequently, the fishery management of *D. maruadsi*.

## Materials & Methods

### Sample collection

**Figure 1:**
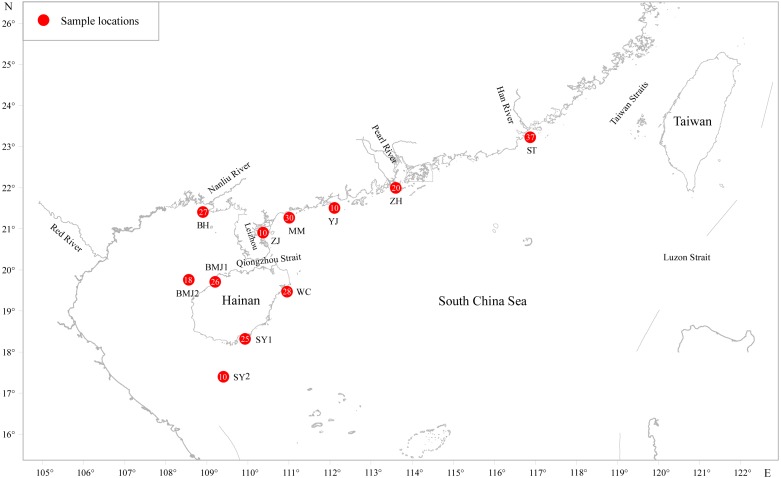
Sampling localities for *D.maruadsi* in the northern South China Sea. Populations are marked by abbreviations and the accompanying pie charts with numbers indicate the number of individuals for each locality.

Two hundred forty-one adult *D. maruadsi* were collected between March 2010 and May 2014 from 11 populations in the northern SCS (Fig. 1 and Table 1) namely, the Shantou inshore water (ST, *N* = 37), the Zhuhai inshore water (ZH, *N* = 20), the Yangjiang inshore water (YJ, *N* = 10), the Maoming inshore water (MM, *N* = 30), the Zhanjiang inshore water (ZJ, *N* = 10), the Wenchang inshore water (WC, *N* = 28), the Sanya inshore water (SY1, *N* = 25), the Sanya offshore water (SY2, *N* = 10), the Baimajing inshore water (BMJ1, *N* = 26), the Baimajing offshore water (BMJ2, *N* = 18), and the Beihai inshore water (BH, *N* = 27). After morphological identification, muscle tissues were taken from individual fish and stored in 95% ethanol at −20 °C until DNA extraction.

**Table 1:** Sampling information and summary of genetic diversity for the mtDNA control region in 11 *D.maruadsi* populations in the northern South China Sea.

Populations	D	N	Hn	*h* ± SD	*π* ± SD	K ± SD	PS
ST	2014.03	37	20	0.946 ± 0.021	0.00392 ± 0.00230	3.252 ± 1.714	23
ZH	2014.01	20	13	0.905 ± 0.054	0.00382 ± 0.00230	3.171 ± 1.712	18
YJ	2014.04	10	9	0.978 ± 0.054	0.00453 ± 0.00282	3.757 ± 2.068	14
MM	2012.05	30	21	0.960 ± 0.022	0.00350 ± 0.00210	2.912 ± 1.572	20
ZJ	2013.07	10	9	0.978 ± 0.054	0.00269 ± 0.00183	2.233 ± 1.341	9
WC	2014.05	28	18	0.960 ± 0.020	0.00404 ± 0.00238	3.350 ± 1.771	22
SY1	2014.04	25	19	0.967 ± 0.024	0.00849 ± 0.00460	7.053 ± 3.426	58
SY2	2010.03	10	7	0.933 ± 0.062	0.00354 ± 0.00229	2.934 ± 1.677	9
BMJ1	2014.04	26	16	0.942 ± 0.029	0.00402 ± 0.00238	3.339 ± 1.770	18
BMJ2	2013.11	18	15	0.980 ± 0.024	0.00468 ± 0.00275	3.881 ± 2.043	23
BH	2013.01	27	17	0.940 ± 0.031	0.00482 ± 0.00277	4.001 ± 2.064	22
Total		241	94	0.953 ± 0.007	0.00443 ± 0.00249	3.691 ± 1.873	91

**Notes.**

D date of collection N sample size Hn number of haplotype *h* ± SD haplotype diversity including standard deviation (SD) *π* ± SD nucleotide diversity including SD K ± SD mean number of pairwise differences including SD PS polymorphic site ST Shantou inshore water ZH Zhuhai inshore water YJ Yangjiang inshore water MM Maoming inshore water ZJ Zhanjiang inshore water WC Wenchang inshore water SY1 Sanya inshore water SY2 Sanya offshore water BMJ1 Baimajing inshore water BMJ2 Baimajing offshore water BH Beihai inshore water

### DNA extraction, PCR, and sequencing

Total genomic DNA was extracted from muscle tissue using the phenol-chloroform protocol (Huang et al., 2002). Polymerase chain reactions (PCRs) for mtDNA control regions were performed in 50 µl containing 39.5 µL dH_2_O, 5 µL 10 × *EasyTaq* buffer, 1 µL dNTP mixture (10 mM), 1 µL forward primer (10 µM, 5′-AACTCCCAAAGCTAGGATTCT-3′), 1 µL reverse primer (10µM, 5′-TTTGTGCTTGCGGGGCTTT-3′) (Niu et al., 2012), 2.5U *EasyTaq* DNA polymerase (TransGen Biotech Co., Ltd., Beijing, China), and 2 µL template DNA (50–100 ng). PCR reactions were performed in a Veriti 96 Well Fast Thermal Cycler (Applied Biosystems, Foster City, CA, USA) under the following parameters: initial denaturation at 94 °C for 5 min followed by 35 cycles of 45 s denaturation at 94 °C, 45 s annealing at 55 °C, and 1 min elongation at 72 °C. The amplification cycle finished with a final elongation at 72 °C for 10 min. All sets of PCR included a negative control reaction tube in which all reagents were included except the template DNA. The PCR products were separated by electrophoresis on 1.2% agarose gel and the *Trans* 2K DNA Marker (TransGen Biotech Co., Ltd., Beijing, China) was used for identifying the target gene size. Successfully amplified PCR products were sent to GenScript Biotech (Nanjing, China) for sequencing.

### Data analysis

Sequences were edited and aligned with the DNASTAR software package (Allex, 1999). All sequences were trimmed using the MEGA 6.06 software (Tamura et al., 2013). Molecular diversity indices, including haplotype diversity (*h*), nucleotide diversity (*π*), the mean number of pairwise differences (*K*), indels, and polymorphic sites, were calculated using the software Arlequin 3.5 (Excoffier & Lischer, 2010). The models of the nucleotide sequence evolution were evaluated by jModelTest v2.1.10 (Darriba et al., 2012), and the Hasegawa-Kishino-Yano (HKY) nucleotide substitution model with a gamma distribution (0.811) and a proportion of invariable sites (0.792) was chosen for the evolutionary analyses.

A Bayesian phylogeny was inferred for all haplotypes under the HKY+G+I model by MrBayes 3.2.6 software (Ronquist et al., 2012). The analysis was run for 10,000,000 generations until the average standard deviation of split frequencies fell below 0.01. The first 25% of burn-in trees were discarded and the subsequent trees were combined to produce a phylogram. Ultimately, a Bayesian inference tree was created and viewed using FigTree 1.4.3 software. The congener, *Decapterus macarellus* (GenBank accession number: KM986880) and *Decapterus macrosoma* (GenBank accession number: KF841444) mtDNA control regions were used as the outgroup. The genealogical relationship among the haplotypes was further evaluated using the media-joining network constructed with Network 5.0.0.3 software (Bandelt, Forster & Rohl, 1999).

Hierarchical analysis of molecular variance (AMOVA) with 10,000 permutations was performed to test the population genetic structure of *D. maruadsi* in the northern SCS. To quantify the genetic divergence, we computed pairwise *F*_ST_ (fixation index) values between populations with 10,000 permutations. An exact test of population differentiation based on haplotype frequencies was also examined with 100,000 steps in the Markov Chain. AMOVA, *F*_ST_ , and the exact test were all calculated using the software Arlequin 3.5.

Historical expansion patterns of *D. maruadsi* for all populations pooled together were calculated using three different approaches. First, Tajima’s *D* and Fu’s *F*s neutrality tests with 10,000 permutations were calculated in Arlequin 3.5 to test the selective neutrality of the *D. maruadsi* control region. Secondly, nucleotide mismatch distribution was performed in Arlequin 3.5 and DnaSP 5.10.01 (Librado & Rozas, 2009) to detect the population expansion history. The fit between the unimodal observed and expected distributions was evaluated by the test statistics of goodness-of-fit, including the sum of squared deviation (SSD) and Harpending’s raggedness index (*Hri*). The statistical significance was estimated with 1,000 bootstrap replicates. Finally, the Bayesian skyline plot was implemented in BEAST 1.7.5 under the HKY+G+I nucleotide substitution model and strict molecular clock model to estimate the change in effective population size across time (Drummond et al., 2012). The Markov Chain Monte Carlo (MCMC) analysis was run for 1 billion generations, yielding high effective sample sizes (ESS) of at least 200, of which the first 10% were discarded as a burn-in. The analysis results were visualized using Tracer 1.7 (Rambaut et al., 2018).

### Molecular rate and its calibration

The molecular rate of control region seems to vary among different bony fishes, such as 3% per myr for rainbow fishes (Zhu et al., 1994), 6.5–8.8% per myr for cichlid fishes (Sturmbauer et al., 2001), and 5–10% per myr for Arctic charr (Brunner et al., 2001). However, there has been no a molecular rate specialized for control region of the fish from Carangidae family. *D. maruadsi* has a small body size, high metabolic rate, and short generation time, suggesting that a rapid molecular clock may be more appropriate for the *D. maruadsi* control region. Thus, a per lineage mutation of 3.75% per myr calculated from the reported control region divergence rates (5–10% per myr) was used for the Bayesian skyline plot analysis of *D. maruadsi* in the present study (Brunner et al., 2001; Liu, 2006; Li et al., 2018).

Several researchers have suggested that it is necessary to calibrate the molecular mutation rate in the analysis of historical population demography (Ho et al., 2005; Ho et al., 2008; Crandall et al., 2012). If the molecular rate is not calibrated on timescales of less than approximately 1–2 Myr before the present, the population expansion time will be overestimated. According to the research results of Ho et al. (2008) and Crandall et al. (2012), 3–10 × faster mutation rates were used to calibrate the population expansion time in the present study. Even so, the evolutionary time estimated based on molecular rate provides an approximate time frame for demographic history.

## Results

### Genetic diversity and haplotype patterns

The partial length sequences of the control region were 828–832 bp. The calculated genetic variations were summarized in Table 1. Sequence alignment revealed 65 transitions, 23 transversions, 11 indels, and 91 polymorphic sites. The *h* of the total population was 0.953, with a range of 0.905–0.980. The *π* and *K* ranged from 0.00269 (ZJ) to 0.00849 (SY1) and from 2.233 (ZJ) to 7.053 (SY1), respectively. This showed that the SY1 population had the highest level of genetic diversity, while the ZJ population had the lowest. The overall *π* and *K* were 0.00443 and 3.691, respectively, indicating low levels of genetic diversity in the *D. maruadsi* population in the northern SCS.

Ninety-one polymorphic sites were observed in the 241 sequences, and defined 94 haplotypes (GenBank accession number: MK294641–MK294734), of which 20 (21.28%) were shared among populations, with a relative frequency ranging from 0.83% to 15.77% (Table 2). For example, H21 (38 specimens, 15.77%) was found in all populations and showed the highest relative frequency among all haplotypes. The main haplotype, H7 (20 specimens, 8.30%), was shared by all populations except the ZJ population. Haplotypes H29 (nine specimens, 3.73%), H57 (18 specimens, 7.47%), H63 (14 specimens, 5.83%), and H74 (16 specimens, 6.64%) occurred in six, eight, seven, and seven populations, respectively. These shared haplotypes also showed different relative frequencies in different populations. The remaining 74 haplotypes (78.72%) were all unique to a single population (private haplotypes), and most of them (71, 95.95%) were singletons.

**Table 2:** Haplotype distributions based on mtDNA control region sequences among 11 *D.maruadsi* populations in the northern South China Sea.

Haplotypes	ST	ZH	YJ	MM	ZJ	WC	SY1	SY2	BMJ1	BMJ2	BH	Total
H7	3	1	2	3		3	2	1	2	2	1	20
H8	3									2		5
H12										1	1	2
H16			1	1		2						4
H21	7	6	1	5	1	4	3	1	2	2	6	38
H29	3			1		1	1		1		2	9
H35			1	1								2
H40						1	1			1	1	4
H45						1			1			2
H50		1		1								2
H51	1			1		2	1				1	6
H55	2		1							1		4
H57	2	3		2	1	1	1		5		3	18
H59	1	1	1							1	1	5
H62				1	1						1	3
H63	3			1			1	2	4	1	2	14
H70						1			1			2
H74	2	1		3	2	3	4	1				16
H81				1	1			2			1	5
H90		1						2				3
PH(IN)	10(10)	6(6)	3(3)	9(9)	4(4)	8(9)	11(11)	1(1)	9(10)	7(7)	6(7)	77
Total	37	20	10	30	10	28	25	10	26	18	27	241

**Notes.**

PH Private haplotypes IN individual number

The PH of ST population are as follows: H22, H27, H43, H65, H77, H82, H85, H88, H89, and H92. The PH of ZH population are as follows: H14, H20, H34, H52, H71, and H79. The PH of YJ population are as follows: H48, H54, and H69. The PH of MM population are as follows: H4, H6, H9, H10, H25, H39, H46, H58, and H75. The PH of ZJ population are as follows: H28, H30, H33, and H73. The PH of WC population are as follows: H5, H15, H36, H47, H64, H67, H83, and H86. The PH of SY1 population are as follows: H2, H19, H24, H37, H38, H53, H61, H78, H84, H93, and H94. The PH of SY2 population is H11. The PH of BMJ1 population are as follows: H1, H3, H13, H18, H26, H32, H44, H60, and H87. The PH of BMJ2 population are as follows: H17, H23, H31, H49, H66, H68, and H72. The PH of BH population are as follows: H41, H42, H56, H76, H80, and H91.

### Phylogenetic relationship

**Figure 2:**
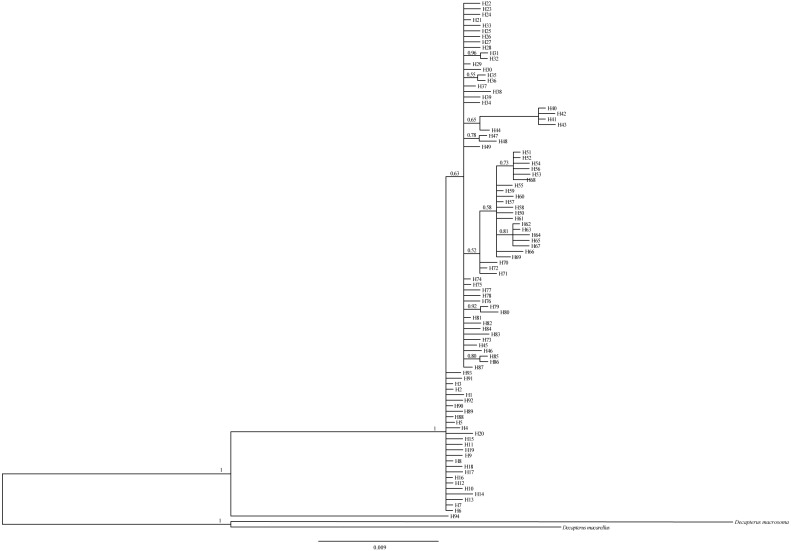
Bayesian inference tree for 94 mtDNA control region haplotypes in *D.maruadsi*. The congener, *Decapterus macarellus* and *Decapterus macrosoma* mtDNA control regions are used as outgroups. The numbers on the branches show the posterior probability values.

**Figure 3:**
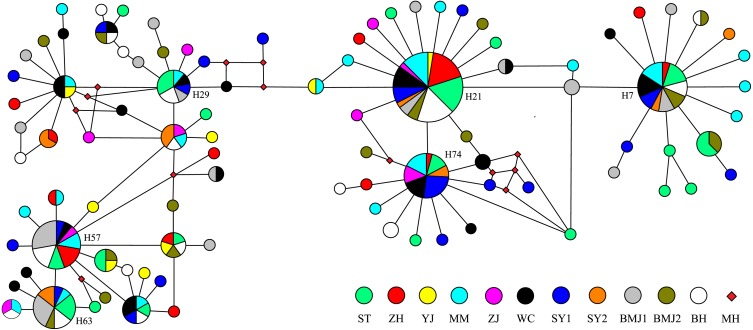
Median-joining network of 94 mtDNA control region haplotypes in *D. maruadsi*. Circles size is proportional to haplotype frequency. MH, Missing haplotypes.

The Bayesian inference tree (Fig. 2) for the 94 haplotypes of *D. maruadsi* showed that haplotypes in every population were scattered throughout the tree and no clustering was observed that corresponded to the sampling localities. A haplotype network (Fig. 3) revealed the presence of six central dominant haplotypes (H7, H21, H29, H57, H63, and H74) at high frequencies across 6–11 populations. All other low-frequency haplotypes radiated in a star-like fashion out from the dominant haplotypes by a few mutational steps. The sharing of interior haplotypes and the endemic distribution of terminal haplotypes indicated the genetic homogeneity among the populations and recent population expansions. This was consistent with the Bayesian inference tree of the 94 haplotypes.

### Genetic structure

Pairwise *F*_ST_ values between populations ranged from −0.053 (between YJ and BMJ2) to 0.058 (between ZJ and SY2) and were statistically non-significant (Table 3). Hierarchical AMOVA results (Table 4) revealed most of the genetic variations (>99%) were attributable to the variations among individuals within populations and only a small amount of the genetic variations (<1%) were explained by variations among geographical groups and among populations within groups. The *F*_CT_, *F*_ST_, and *F*_SC_ values of hierarchical AMOVA analysis were all low and not statistically significant. The results demonstrated that no significant genetic differentiation existed among populations. The finding could be further supported by the results of the exact tests among the population which revealed no significance (exact *P* value = 0.0986 ± 0.0517).

**Table 3:** Matrix of pairwise *F*_ST_ (below diagonal) and *P* values (upper diagonal) between populations.

Populations	ST	ZH	YJ	MM	ZJ	WC	SY1	SY2	BMJ1	BMJ2	BH
ST		0.703	0.909	0.941	0.377	0.910	0.510	0.626	0.617	0.976	0.454
ZH	−0.016		0.863	0.661	0.667	0.627	0.680	0.258	0.596	0.503	0.607
YJ	−0.041	−0.044		0.809	0.627	0.904	0.929	0.467	0.898	0.969	0.795
MM	−0.019	−0.016	−0.037		0.356	0.996	0.507	0.475	0.505	0.912	0.226
ZJ	−0.000	−0.026	−0.023	0.003		0.530	0.766	0.160	0.199	0.271	0.615
WC	−0.019	−0.013	−0.040	−0.026	−0.010		0.877	0.585	0.367	0.951	0.393
SY1	−0.004	−0.010	−0.041	−0.004	−0.030	−0.014		0.498	0.263	0.840	0.596
SY2	−0.020	0.020	−0.021	−0.011	0.058	−0.017	−0.010		0.381	0.701	0.203
BMJ1	−0.011	−0.015	−0.044	−0.008	0.028	−0.023	0.006	−0.002		0.768	0.305
BMJ2	−0.029	−0.010	−0.053	−0.026	0.015	−0.030	−0.017	−0.030	−0.023		0.575
BH	−0.003	−0.012	−0.033	0.012	−0.019	0.000	−0.007	0.026	0.006	−0.011	

**Table 4:** Analyses of molecular variance (AMOVA) for testing the genetic subdivision of 11 *D.maruadsi* populations using mtDNA control region sequences across geographic districts.

	d.f.	Sum of squares	Variance components	Percentage of variation	Fixation indices	Significance tests
Groups: Leizhou Peninsula and Hainan Island (ST, ZH, YJ, MM, ZJ, WC, SY1, SY2) (BMJ1, BMJ2, BH)						
Among groups	1	1.917	0.007 Va	0.37	*F*_CT_ = 0.004	*p* = 0.156 ± 0.004
Among populations within groups	9	11.968	−0.025 Vb	−1.37	*F*_SC_ = − 0.014	*p* = 0.946 ± 0.002
Within populations	230	428.983	1.865 Vc	101	*F*_ST_ = − 0.010	*p* = 0.933 ± 0.002
Groups: Pearl River, Qiongzhou Strait, Leizhou Peninsula (ST) (ZH, YJ, MM, ZJ)(WC, SY1, SY2)(BMJ1, BMJ2, BH)						
Among groups	3	4.287	0.004 Va	0.24	*F*_CT_ = 0.002	*p* = 0.304 ± 0.005
Among populations within groups	7	9.598	−0.026 Vb	−1.4	*F*_SC_ = − 0.014	*p* = 0.8820 ± 0.003
Within populations	230	428.983	1.865 Vc	101.16	*F*_ST_ = − 0.012	*p* = 0.934 ± 0.002
Groups: no genetic barriers (ST, ZH, YJ, MM, ZJ, WC, SY1, SY2, BMJ1, BMJ2, BH)						
Among populations	10	13.682	−0.022 Va	−1.2		
Within populations	230	422.279	1.836 Vb	101.20	*F*_ST_ = − 0.012	*p* = 0.933 ± 0.003

**Figure 4:**
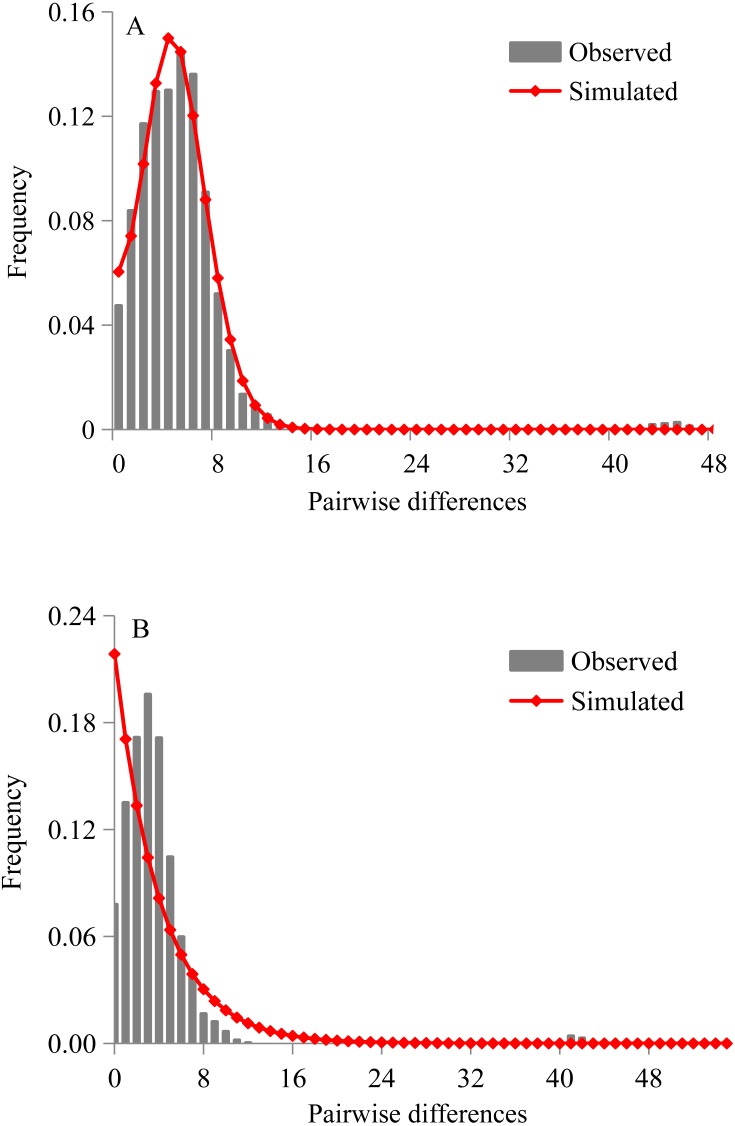
Mismatch distribution analyses of *D. maruadsi* for mtDNA control region. (A) Sudden expansion model. (B) Constant population size model.

### Demographic history

Both Tajima’s *D* statistics (Tajima’s *D* =  − 2.201, *p* = 0.0005) and Fu’s *F*s test (Fu *F*s = −25.180, *p* = 0.0002) were negative and highly significant in pooled samples. The mismatch distributions of *D. maruadsi* for all populations pooled together showed a unimodal pattern under the sudden expansion model (Fig. 4A) and constant population size model (Fig. 4B). Under the sudden expansion model, the observed distributions were included in the 90%, 95%, and 99% confidence intervals obtained by simulated distribution. The SSD values (SSD = 0.001, *p* = 0.840) and *Hri* (*Hri* = 0.007, *p* = 0.936) were small and statistically non-significant, which showed no significant deviation between the observed distributions and expected distributions. Under the constant population size model, a poor goodness-of-fit was found between the observed and expected distributions. Both the neutrality test and mismatch distribution analyses suggested a recent population demographic expansion for *D. maruadsi* populations in the northern SCS. However, a low migrants per generation (M < 50) was detected under the spatial expansion model. This may lead to a larger variance of mismatch distribution and incorrectly estimating the parameters for spatial expansion (Ray, Currat & Excoffier, 2003; Excoffier, 2004). Therefore, the spatial expansion model was not chosen to analyze the range expansion history of *D. maruadsi*.

**Figure 5:**
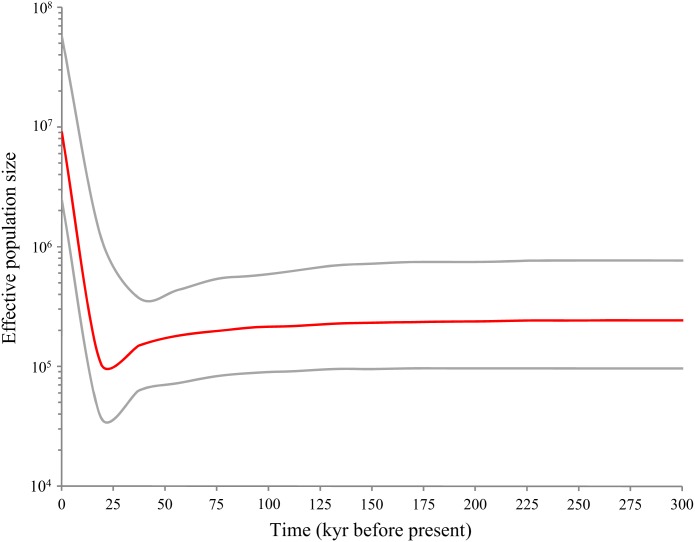
Bayesian skyline plots (BSP) based on mtDNA control region of *D. maruadsi* from the northern South China Sea. The figure shows effective population size (*Ne*) changes of *D. maruadsi* through time. Intermediate red line represents the median estimate of *Ne*. The gray lines show upper and lower 95% highest posterior density (HPD) limits of *Ne*.

Bayesian skyline plot (Fig. 5) revealed a pattern of glacial decline and postglacial growth in the effective population size based on the mutation rate of 3.75% per myr. The population decline happened about 18,000–37,000 years before present (YBP) that coincided with the LGM. Following the LGM and continuing to the present (0–18,000 YBP), *D. maruadsi* has undergone a rapid episode of population growth, which resulted in an about 100-fold increase in the maximum effective population size. The results suggested that *D. maruadsi* in the northern SCS experienced demographic expansion after the LGM. Use of the 3–10 ×faster mutation rates shortened the timing of population expansion and the calibrated time was 1,800–6,000 YBP, which further confirmed that *D. maruadsi* in the northern SCS experienced a recent population expansion during the Holocene.

### Artificial statistical bias adjacent geographical populations

To detect artificial statistical bias due to a low sample size from YJ, ZJ, and SY2 populations, some adjacent geographical populations were combined into three geographic groups for genetic differentiation analysis, including the western Guangdong group (WGD: ZH, YJ, MM, ZJ), Sanya group (SY: SY1, SY2), and Baimajing group (BMJ: BMJ1, BMJ2). Genetic homogeneity was demonstrated by AMOVA (Table 5), pairwise *F*_ST_ (*F*_ST_ =  − 0.0189–0.00674, *P* = 0.235 − 0.992), and exact tests (exact *P* value = 0.178 ± 0.0758) among three geographic groups and the other three populations (ST, WC, BH). This indicated that low sample sizes for YJ, ZJ, and SY2 have not influenced the results of this study.

**Table 5:** Analyses of molecular variance (AMOVA) for testing the genetic subdivision of three geographic groups (WGD, SY, BMJ) and the other three populations (ST, WC, BH) using mtDNA control region sequences.

	d.f.	Sum of squares	Variance components	Percentage of variation	Fixation indices	Significance tests
Groups: Leizhou Peninsula and Hainan Island (ST, WGD, WC, SY) (BMJ, BH)						
Among groups	1	1.938	0.00562 Va	0.30	*F*_CT_ = 0.003	*p* = 0.198 ± 0.004
Among populations within groups	4	5.598	−0.0117 Vb	−0.63	*F*_SC_ = − 0.006	*p* = 0.805 ± 0.004
Within populations	235	435.334	1.852 Vc	100.33	*F*_ST_ = − 0.003	*p* = 0.755 ± 0.004
Groups: Pearl River, Qiongzhou Strait, Leizhou Peninsula (ST) (WGD)(WC, SY)(BMJ, BH)						
Among groups	3	4.307	−0.002 Va	−0.09	*F*_CT_ = − 0.001	*p* = 0.619 ± 0.005
Among populations within groups	2	3.228	−0.007 Vb	−0.40	*F*_SC_ = − 0.004	*p* = 0.725 ± 0.004
Within populations	235	435.334	1.852 Vc	100.49	*F*_ST_ = − 0.005	*p* = 0.759 ± 0.005
Groups: no genetic barriers (ST, WGD, WC, SY, BMJ, BH)						
Among populations	5	7.535	−0.00883 Va	−0.48		
Within populations	235	435.334	1.852 Vb	100.48	*F*_ST_ = − 0.00479	*p* = 0.752 ± 0.004

## Discussion

### Demographic history

This study revealed *D. maruadsi* in the northern SCS experienced a rapid demographic expansion after a period of low effective population size during the Holocene. The results suggested better conditions (e.g., the enlargement of habitat and the significant increase of paleo-productivity) after the LGM driving the population expansion of *D. maruadsi*.

During the LGM, the sea-level dropped 100–120 m in the South China Sea (Wang & Sun, 1994). The dropping of the sea-level exposed shallow coastal continental shelves and turned the SCS into a semi-closed land-locked sea (about one-fifth smaller than it is now) (Wang & Sun, 1994; Wang, 1999), greatly reducing the habitat and population abundance of many marine fishes in the continental shelf (He et al., 2015; Zhu, Cheng & Rogers, 2016). *D. maruadsi* widely distributes in the northern shallow continental shelf of the SCS within 180 m and its spawning grounds are located mainly in shallow nearshore waters less than 120 m (Deng & Zhao, 1991). With the falling sea-level during the LGM, most of its spawning grounds were exposed and gradually moved from shallow-water to the deeper water. Thus, *D. maruadsi* should have undergone intensive spawning ground loss and change, which may have caused a decrease in the effective population size, abundance, and distribution. The surviving *D. maruadsi* had to contract into the potential glacial refugia (semi-closed SCS). However, the subsequent rising sea-level of the postglacial-Holocene provided new niche space for *D. maruadsi*, leading to sustained and rapid growth in the effective population size of this species. Similar patterns of population dynamics have been revealed for some marine fishes in response to the postglacial rising sea-level in the northern SCS (Liu et al., 2006; Xiao et al., 2014; Gao et al., 2018).

The paleo-productivity of the northern SCS was mainly influenced by the fluctuant East Asian monsoon and Kuroshio Current (Wang, 1999; Yu & Qiu, 2016). After the LGM, the East Asian summer monsoon gradually strengthened, which caused high precipitation and increased land-source influx into the northern SCS (Jian, Wang & Kienast, 1999). Accordingly, high levels of terrigenous nutrients from the Asian continent were transported into the northern SCS ecosystems through the Pearl River, Han River, Nanliu River, Red River, and other sources of fluvial runoff (Jian et al., 1999; Gao, 2013; Qu & Huang, 2019). Moreover, the enhanced summer monsoon could drive upwelling containing high nutrient input into the northern SCS (Jian, Wang & Kienast, 1999). Obviously, the enhanced summer monsoon during the deglaciation period increased the nutrient input of the northern SCS, which led to higher paleo-productivity than that of the LGM. During the LGM, the Kuroshio Current was forced eastward due to the formation of a land bridge between Taiwan and the Ryukyu Islands, which prohibited its flow into the SCS (Ujiié et al., 2003). By contrast, the postglacial Kuroshio Current branches into the northern SCS through the Luzon Strait along with the rise of the sea-level (Nan, Xue & Yu, 2015). The Kuroshio intrusion induces upwelling and meso- and micro-scale eddies, strengthening the vertical mixing of the water layer and nutrient supplies to the upper ocean (Yu & Qiu, 2016). Additionally, the Kuroshio intrusion could transport large amounts of nutrients into the northern SCS (Xu et al., 2018), promoting the increase of paleo-productivity in the northern SCS. The combination of the above factors strongly prove that the northern SCS had significantly higher paleo-productivity during deglaciation (> 30 gC/(m^2^⋅10^3^a)) than the LGM (< 25gC/(m^2^⋅10^3^a)) (Kuhnt, Hess & Jian, 1999; Zhao & Wang, 1999). The significant increase of paleo-productivity in the northern SCS after the LGM has a close correlation to the recent rapid population growth of *D. maruadsi*.

All in all, LGM-Holocene climate oscillations gave rise to drastic changes in the East Asian monsoon circulation, sea-level, ocean current patterns, and upwelling density of the western Pacific marginal seas (Wang, 1999; Jian, Wang & Kienast, 1999; Ujiié et al., 2003). These changes resulted in enlargement of nearshore habitat and significant increase of primary productivity, which were believed to be the main cause of the rapid demographic expansion of *D. maruadsi*. Similar phenomena have been reported in other fish species, such as *Pampus echinogaster* (Li et al., 2018) and *Trichiurus japonicus* (He et al., 2014). However, it is not yet possible to discriminate the relative impact of changes in habitat or paleo-productivity based on the existing mtDNA data.

### Population structure

In this study, low levels of genetic diversity and high levels of genetic homogeneity were detected in the *D. maruadsi* population from the northern SCS. Contemporary genetic diversity and structure is influenced by demographic history (Hewitt, 1996; Hewitt, 2000). The rapid population growth after a period of low effective population size could enhance the retention of new mutations, but it might not accumulate enough nucleotide diversity in the short term (Avise, Neigel & Arnold, 1984; Rogers & Harpending, 1992). Therefore, the population decline and the subsequent rapid population expansion may lead to the loss of diversity and explain the low levels of genetic diversity in *D. maruadsi* in the northern SCS. The recent rapid population expansion also indicates that *D. maruadsi* in the northern SCS is far from equilibrium due to the lack of sufficient evolutionary time (Slatkin, 1993), which should be an important historical reason for genetic homogeneity among *D. maruadsi* populations. A similar genetic structure and explanation was found in *E. japonicus* (Liu et al., 2006) and *Pennahia argentata* (Han et al., 2008) along the coastal waters of China.

Several other factors aside from the historical process may have also led to the lack of significant genetic differentiation among *D. maruadsi* populations in the northern SCS. First, the planktonic eggs and larvae of *D. maruadsi* (Deng & Zhao, 1991) could disperse on ocean currents to facilitate genetic exchange over great distances. There are many annual or semi-annual ocean currents in the northern SCS. The northward currents, such as the southwest monsoon drift, South China Sea warm current, the coastal current of eastern Guangdong, and the coastal current of eastern Hainan (Qiao, 2012), are beneficial for the northward dispersal of the southern groups (e.g., SY1, ZJ, and MM populations). The southward currents, including the northeast monsoon drift, Guangdong coastal current, and the Kuroshio branch in South China Sea (Li, Zhao & Wasiliev, 2000), promote populations from the eastern Guangdong to migrate south. Furthermore, *D. maruadsi* pelagic larvae on both sides of Leizhou Peninsula and Hainan Island could cross the Qiongzhou Strait for genetic exchange along the residual current in the Qiongzhou Strait (Gao, 2013). The planktonic eggs and larvae of *D. maruadsi* may travel great distances on ocean currents in the northern SCS, which may be sufficient to produce genetic homogeneity. Secondly, adult *D. maruadsi* from the northern SCS spawn throughout the year, and the spawning ground spreads from the Taiwan shoal to Beibu Gulf (Deng & Zhao, 1991). The overlapping spawning time and spawning ground could maintain genetic connectivity amid geographic separation.

The lack of significant genetic structure implies that *D. maruadsi* in the northern SCS should be considered a single panmictic population. However, some significant biological differences are found among the different populations of *D. maruadsi* in the northern SCS. The average fork length and body weight of *D. maruadsi* in Beibu Gulf and its mouth are 145 mm (dominant fork length = 110–160 mm) and 42 g (dominant body weight = 20–60 g), respectively; while those in the eastern Hainan Island are 158.7 mm (dominant fork length = 130–220 mm) and 102.5 g (dominant body weight = 70–140 g), respectively (Jia, Li & Qiu, 2005). Additionally, different peak spawning periods are found among the various geographical populations of *D. maruadsi* in the northern SCS (Deng & Zhao, 1991). For example, the peak spawning season for the Beibu Gulf population is from January to March, from May to June for the population from southeast Hainan Island, and from February to April for the populations from the Pearl River estuary to east Guangdong (Jia, Li & Qiu, 2005). The potential mismatch between the population’s genetic structure (genetic homogeneity) and biological characteristics (significant difference) reminds us to identify and manage *D. maruadsi* populations carefully. However, owing to maternal inheritance, the mtDNA control region sequence provides no information about male migration and has some limitations in respect of fishery management (Waples, Punt & Cope, 2008). Thus, future integrative studies based on nuclear markers with biparental inheritance (such as microsatellites and SNPs), and biological and ecological characteristics are needed for more accurate identification of the population structure and MUs of *D. maruadsi* in the northern SCS.

## Conclusions

The present study reveals that LGM-Holocene climate oscillations had an important impact on the demographic history, genetic structure, and genetic diversity of *D. maruadsi* in the northern SCS. LGM-Holocene climate change gave rise to drastic changes in the habitat and paleo-productivity, which resulted in the recent rapid population expansion. The demographic history is considered to be responsible for the low levels of genetic diversity for *D. maruadsi* in the northern SCS. The low genetic diversity suggests that the environmental adaptive capacity and evolutionary potential of *D. maruadsi* may decline from a long-term evolutionary perspective. This reminds us to strengthen fishery resource management and protection in order to sustain the productivity of *D. maruadsi* in the northern SCS. Moreover, no significant genetic differentiation was detected among the *D. maruadsi* populations, which has a close correlation to recent rapid population expansion, the dispersion of planktonic eggs and larvae on ocean currents, and overlapping spawning time and spawning grounds. However, owing to the potential mismatch between the population’s genetic structure and biological characteristics and some accuracy limitations of mtDNA to the appropriate identification of the population structure and MUs, the lack of significant genetic structure does not mean that *D. maruadsi* in the northern SCS should be managed as a single unit.

##  Supplemental Information

10.7717/peerj.7953/supp-1 Supplemental Information 1 241 sequences from 11 populations in the northern SCS

10.7717/peerj.7953/supp-2 Supplemental Information 2 94 haplotypes sequences and accession numbers

## Additional Information and Declarations

### Field Study Permissions

The following information was supplied relating to field study approvals (i.e., approving body and any reference numbers): The fish used in the study were collected by fishermen. All fish were dead when received, so this study does not require ethical approval.

### Data Availability

The following information was supplied regarding data availability: The 94 haplotype sequences are available at GenBank: MK294641 to MK294734.
